# Accurate calculation of side chain packing and free energy with applications to protein molecular dynamics

**DOI:** 10.1371/journal.pcbi.1006342

**Published:** 2018-12-27

**Authors:** John M. Jumper, Nabil F. Faruk, Karl F. Freed, Tobin R. Sosnick

**Affiliations:** 1 Department of Biochemistry and Molecular Biology, University of Chicago, Chicago, Illinois, United States of America; 2 Department of Chemistry, and The James Franck Institute, University of Chicago, Chicago, Illinois, United States of America; 3 Graduate Program in Biophysical Sciences, University of Chicago, Chicago, Illinois, United States of America; 4 Institute for Biophysical Dynamics, University of Chicago, Chicago, Illinois, United States of America; Fox Chase Cancer Center, UNITED STATES

## Abstract

To address the large gap between time scales that can be easily reached by molecular simulations and those required to understand protein dynamics, we present a rapid self-consistent approximation of the side chain free energy at every integration step. In analogy with the adiabatic Born-Oppenheimer approximation for electronic structure, the protein backbone dynamics are simulated as preceding according to the dictates of the free energy of an instantaneously-equilibrated side chain potential. The side chain free energy is computed on the fly, allowing the protein backbone dynamics to traverse a greatly smoothed energetic landscape. This computation results in extremely rapid equilibration and sampling of the Boltzmann distribution. Our method, termed *Upside*, employs a reduced model involving the three backbone atoms, along with the carbonyl oxygen and amide proton, and a single (oriented) side chain bead having multiple locations reflecting the conformational diversity of the side chain’s rotameric states. We also introduce a novel, maximum-likelihood method to parameterize the side chain interactions using protein structures. We demonstrate state-of-the-art accuracy for predicting *χ*_1_ rotamer states while consuming only milliseconds of CPU time. Our method enables rapidly equilibrating coarse-grained simulations that can nonetheless contain significant molecular detail. We also show that the resulting free energies of the side chains are sufficiently accurate for *de novo* folding of some proteins.

## Author summary

To address the large gap between time scales that can be easily reached by molecular simulations and those required to understand protein dynamics, we propose a new methodology that computes a self-consistent approximation of the side chain free energy at every integration step. As a result, the method largely eliminates side chain friction, a factor that greatly slows all atom approaches. With this speed-up, our method is capable of folding some proteins in CPU-hours. We also demonstrate state-of-the-art accuracy for predicting *χ*_1_ rotamer states with a 100-fold speedup. Because conformational space can be rapidly explored with our inexpensive method, it is an excellent starting point for studying protein dynamics involving large conformational transitions.

## Introduction

Two major challenges must be overcome in order to accurately simulate protein dynamics. The first is the necessity of balancing the large and competing sources of energy and entropy whose sum determines both the thermodynamics and the native conformation of the protein. The second challenge involves the intensive sampling required to obtain a Boltzmann ensemble of conformations. The sampling challenge is addressed here by integrating out the side chain degrees of freedom to produce a coarse-grained configuration defined just in terms of the backbone N, C_*α*_, and C atoms. Consequently, backbone motions evolve on a smoother free energy surface with greatly reduced side chain rattling (molecular friction) compared to that for standard all-atom molecular dynamics simulations.

The uncertainty in the position of coarse-grain interactions heightens the difficulty of accurately parameterizing a coarse-grained model. We do not follow the customary process of matching the energies of the coarse-grained model to approximate the already inexact energies of atomistic force fields or try to interpret raw statistics for the distribution of interatomic distances in the Protein Data Bank (PDB) [1] along with a reference state [2]. Instead, our side chain interaction energies are determined as those that best reproduce the side chain conformations observed in the PDB, given the native-state backbone configurations. That is, we search for an energy function that assigns on average the highest probability to the native *χ*_1_ rotamer.

This maximum-likelihood approach has key advantages: 1. It directly provides an interpretation of the structural information as a sample from the statistical mechanical ensemble of side chain packing, and 2. it can be evaluated quickly since we show that approximating the Boltzmann distribution for the side chains in a fixed backbone configuration does not require laborious Monte Carlo sampling of the *χ* angles in the side chain.

Using our side chain ensembles, we are able to predict *χ*_1_ rotamer configurations with similar accuracy as SCWRL4 [3] and OSCAR [4] [5], yet our predictions take less than 1% of the computational time. We are also exceed the speed of the rapid side chain packing algorithm RASP [6] by more than an order of magnitude. The accuracy of our side chain rotamer predictions validates that our side chain interaction potential captures much of the important physics of side chain interactions, suggesting suitability for molecular dynamics.

## Methods

### *Upside* model

**Fig 1:**
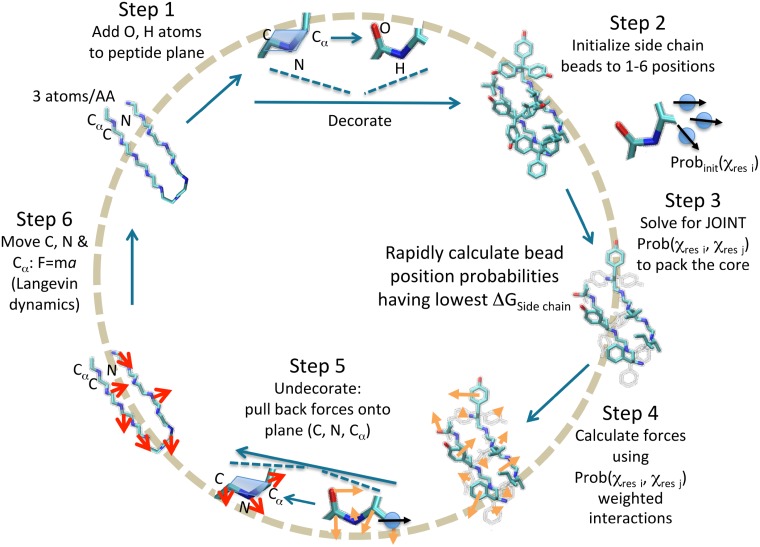
Six step inner loop of *Upside* calculation. The side chain potential enters into the integration step simply as a complicated, many-body energy function that may be treated with standard techniques of molecular simulations.

**Fig 2:**
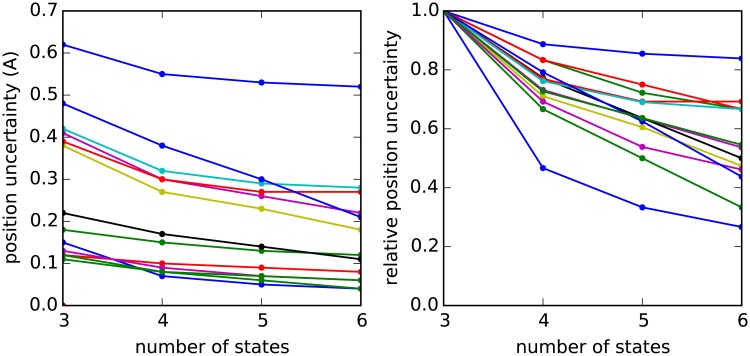
Error in the position as a function of the number of side chain states, resulting from a decomposition of rotamer states into coarse-grained states. The table summarizes the number of states chosen for each amino acid type. The relative uncertainty is the positional uncertainty for each number of states divided by the accuracy at three states. One, three, or six rotamer states are used, depending on the residue type. For residues without a rotatable *χ*_2_, such as valine, only three states are needed. The time to compute the pairwise interactions and solve for the free energy scales roughly as the number of coarse rotamer states squared, so the use of fewer coarse states is preferred. Ile, Leu and Lys are the three residues with rotatable *χ*_2_ where only 3 states are assigned.

**Fig 3:**
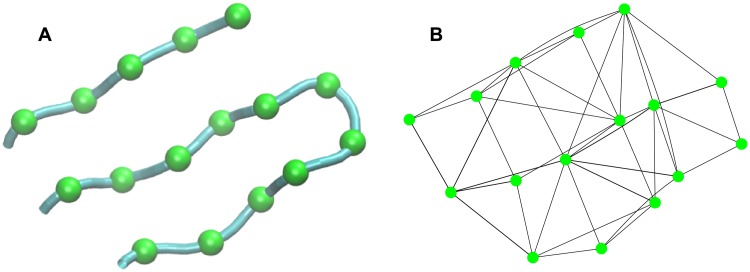
Fragment of protein G with associated interaction graph (*R*_cutoff_ = 7Å). A pair of residues is assigned a connection whenever their side chain beads are within *R*_cutoff_ for any side chain states.

The strategy in our *Upside* model is to perform dynamics simulations for just the N, C_*α*_, and C atoms that define the backbone trace, while still including sufficient structural detail (side chain structures and free energies, etc.) necessary to compute realistic forces. Fig 1 presents an overview of the six step computational cycle used for molecular (Langevin) dynamics simulations. While the overarching goal of our work is extremely rapid molecular dynamics, our new interaction model gives very accurate and rapid predictions of side chain *χ*_1_ angles. The inclusion of the side chain free energy, rather than the side chains themselves, greatly smooths the potential governing the dynamics of the backbone trace, especially because of the reduction of steric rattling. The parameters used in the energy calculation are trained to maximize the probability of the average side chain having the native *χ*_1_. The major computational steps are:

Step 1 The loop begins (upper left corner) with each residue in the protein being represented with 3 backbone atoms, the N, C_*α*_ and C. Based on the position of these atoms, the carbonyl oxygen, O, and amide proton, H, are deterministically placed.
Step 2 Each side chain, represented by a single oriented bead, is assigned an initial probability for being in 1–6 states, depending on the residue type (Fig 2) and the average frequency observed in the PDB. The state of the bead is defined by its position and an orientation, (x,y,z,v), where v is a unit vector relative to the peptide plane. The position and orientation of the bead define the interaction graph (Fig 3).
Step 3 The pair-wise state probabilities of all side chains are simultaneously and rapidly calculated using belief propagation to produce the lowest system free energy satisfying Eq 7.
Step 4 Forces on the 3 backbone atoms, as well as on the O, H and side chain beads are calculated from the derivative of the free energy.
Step 5 Forces on the O, H and beads are “pulled back” and added to the forces on the 3 backbone atoms by reversing the placement process via Eq 16.
Step 6 Langevin dynamics (implicit solvent with friction) are run on the 3 backbone atoms using the forces calculated in Steps 4 and 5.

Below we describe the steps in detail.

### Side chain free energy evaluation

One can consider a representation of the protein in terms of the coordinates ({*b*_*i*_}, {*χ*_*i*_}) where *b*_*i*_ represents the positions of the backbone N, C_*α*_, and C atoms on the *i*-th residue and *χ*_*i*_ represents the side chain *χ*-angles on the *i*-th residue. Because bond lengths and angles are relatively constant, the positions of the atoms can be reconstructed with high accuracy from the ({*b*_*i*_}, {*χ*_*i*_}) coordinates (Step 1). Given a potential energy *V*({*b*_*i*_}, {*χ*_*i*_}), we calculate the free energy as a function of the backbone configuration, from the logarithm of the partition function

$$\bar{V}\left(\left\{b_{i}\right\}\right)=-\text{log}\int d\chi_{1}\;\cdots \chi_{N}\;e^{-V(\left\{b_{i}\right\},\left\{\chi_{i}\right\})}.$$ (1)

Natural energy units are used with *k*_B_*T* = 1. An intermediate step of this derivation requires a discrete approximation $\left\{{\tilde{\chi }}_{i}\right\}$ for our *χ*-angles and a discrete approximation $\bar{V}\left(\left\{b_{i}\right\},\left\{{\tilde{\chi }}_{i}\right\}\right)$ for the potential.

Rather than directly calculate Eq 1, we define an intermediate discrete approximation to $\bar{V}$ where the side chain bead positions and orientations are defined to be in up to six discrete positions that are amenable to approximation techniques (Step 2). This discretization process is accomplished using a discrete coarse-graining function *g* which maps the continuous side chain rotamers *χ*_*i*_: ${\tilde{\chi }}_{i}=g\left(\chi_{i}\right)$, where ${\tilde{\chi }}_{i}$ is a state label (${\tilde{\chi }}_{i}\in \left\{1,\ldots ,6\right\}$ as each side chain is represented by a bead located at one of up to 6 positions). The coarse-grain potential $\tilde{V}$ is defined so that

$$e^{-\tilde{V}\left(\left\{b_{i}\right\},\left\{{\tilde{\chi }}_{i}\right\}\right)}\approx \int d\chi_{1}\;\cdots \chi_{N}\;(\prod_{i}\delta_{{\tilde{\chi }}_{i}g\left(\chi_{i}\right)})e^{-V(\left\{b_{i}\right\},\left\{\chi_{i}\right\})}.$$ (2)

**Fig 4:**
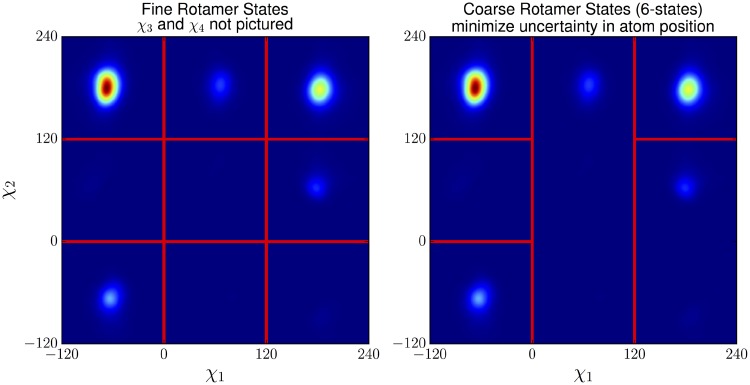
Example of optimized coarse states for arginine overlaid on the PDB distribution of the rotamer angles *χ*_1_ and *χ*_2_. Each of the six coarse states contains only a single fine state that has high probability, so that the variance of dihedral angles within each coarse state is small.

In principle, any coarse-grain function for the side chains may be used. The discrete form $\tilde{V}$ of the potential provides an accurate approximation as the distribution of *χ*-angles is sharply peaked (in the true potential *V*) within each discrete state $\tilde{\chi }$. Fig 4 provides an example of a function while Subsection **Optimized mapping to coarse states** shows how the optimized function *g* is derived.

We make the following assumptions on the form of $\tilde{V}$. First, we assume an explicit function $y_{i}\left(b_{i},{\tilde{\chi }}_{i}\right)$ exists for the side chain coordinates based only on the backbone coordinates and side chain state for residue *i*. We may relax the requirement to consider a single residue’s backbone position, but it is required that *y*_*i*_ depend on only a single side chain *state* ${\tilde{\chi }}_{i}$. These directed coordinates are approximately the side chain centers of mass with direction given by the C_*β*_−C_*γ*_ bond vector.

The $\tilde{V}$ are expressed as the sum of a backbone term involving dihedral angle preferences, side chain-backbone interactions (including hydrogen bonding), and pairwise interactions involving the side chains,

$$\begin{array}{c} \tilde{V}\left(\left\{b_{i}\right\},\left\{{\tilde{\chi }}_{i}\right\}\right)=V^{\text{backbone}}\left(\left\{b_{k}\right\}\right)+\\ \sum_{i}V_{i}^{\left(1\right)}\left(\left\{b_{k}\right\},{\tilde{\chi }}_{i},y_{i}\left(b_{i},{\tilde{\chi }}_{i}\right)\right)+\\ \sum_{i,j}V_{ij}^{\left(2\right)}\left(y_{i}\left(b_{i},{\tilde{\chi }}_{i}\right),y_{j}\left(b_{j},{\tilde{\chi }}_{j}\right)\right), \end{array}$$ (3)

The pair interaction $V_{ij}^{\left(2\right)}\left(y_{i},y_{j}\right)=0$ for the side chain is taken to vanish beyond a cutoff *R*_cutoff_. The dependence of the potential on the backbone is completely general, but the potential is assumed to contain at most a pairwise dependence on the discrete rotamer states ${\tilde{\chi }}_{i}$. Explicit parameterizations for *y*_*i*_ and $\tilde{V}$ are defined in the **Subsection Bead locations and interactions** using the principle of maximum likelihood.

One can simulate the Boltzmann ensemble for $\tilde{V}$ using molecular dynamics for the backbone {*b*_*i*_} and Monte Carlo moves for the side chain states $\left\{{\tilde{\chi }}_{i}\right\}$. But the strong steric interactions are likely to lead to slow equilibration and dynamics for both the side chains and backbone. Because we are predominantly interested in backbone motions, we return to the free energy $\bar{V}$ in Eq 1, now summing over discrete side chain states instead of integrating over continuous side chain angles,

$$e^{-\bar{V}\left(\left\{b_{i}\right\}\right)}\approx \sum_{{\tilde{\chi }}_{1},\ldots ,{\tilde{\chi }}_{N}}e^{-\tilde{V}\left(\left\{b_{i}\right\},\left\{{\tilde{\chi }}_{i}\right\}\right)}.$$ (4)

The potential $\bar{V}\left(\left\{b_{i}\right\}\right)$ represents a further coarse-graining of the system by completely replacing the influence of the side chains with a potential describing their adiabatic free energy for a given fixed backbone conformation. Because $\bar{V}$ depends only on the (continuous) backbone coordinates, this choice of $\bar{V}$ enables running standard molecular dynamics simulations instead of a hybrid of Monte Carlo and molecular dynamics.

Importantly, the potential $\bar{V}\left(\left\{b_{i}\right\}\right)$ is a much smoother function of the backbone coordinates than the original *V*({*b*_*i*_}, {*χ*_*i*_}) because the replacement of the side chain degrees of freedom with the approximate free energy of the side chains greatly reduces steric rattling and molecular friction. The reduction of the ruggedness of the energy landscape enhances diffusion within conformational basins but preserves the overall structure and barriers that define the conformational ensemble.

### Approximating the side chain free energies

The benefits of running dynamics with our coarse grained $\bar{V}$ could enter at great cost because using even with three coarse-grained states per side chain, there are over 3^*N*^ $\tilde{\chi }$-states in Eq 4. However, the vast majority of those 3^*N*^ states have steric clashes or other large energies and, therefore, contribute little to the side chain free energy. In this section, we describe how we take advantage of this potentially huge reduction in relevant states to calculate an approximate side chain free energy.

To approximate the free energy of the side chains $\bar{V}$, we express the problem in the language of Ising models so that we can apply standard techniques developed in that context. For a fixed backbone configuration {*b*_*i*_},

$$\begin{array}{cc} \tilde{V}\left(\left\{b_{i}\right\},\left\{{\tilde{\chi }}_{i}\right\}\right) & =\bar{v}\left(\left\{{\tilde{\chi }}_{i}\right\}\right)\\ & =\sum_{i}v_{i}^{\left(1\right)}\left({\tilde{\chi }}_{i}\right)+\sum_{\begin{array}{c} i,j\\ \text{neighbors} \end{array}}v_{ij}^{\left(2\right)}\left({\tilde{\chi }}_{i},{\tilde{\chi }}_{j}\right), \end{array}$$ (5)

where the potentials $\bar{v}$ are written in lowercase to indicate suppression of the dependence on the fixed backbone coordinates {*b*_*i*_} in order to focus on the side chain contribution. Notice that with the backbone positions fixed, each single-residue potential $v_{i}^{\left(1\right)}$ is simply a vector with as many components as the number of possible states for ${\tilde{\chi }}_{i}$ (e.g. length-6 vectors). Similarly, each of the pair potentials $v_{ij}^{\left(2\right)}$ is a small 6x6 matrix of potential energies to cover a maximum of 36 possibilities. These single and pair potentials are calculated only once before evaluating the free energy as described in Subsection **Bead locations and interactions**. Moreover, the pair summation in Eq 5 only applies for residues pairs *i* and *j* that are neighbors spatially. A pair of residues (*i*, *j*) are neighbors if inter-residue distance $|y_{i}\left({\tilde{\chi }}_{i}\right)-y_{j}\left({\tilde{\chi }}_{j}\right)|$ is less than a cutoff *R*_cutoff_ for any of their possible discrete states $\left({\tilde{\chi }}_{i},{\tilde{\chi }}_{j}\right)$. In this work, we use *R*_cutoff_ = 7 Å for side chain-side chain interactions and *R*_cutoff_ = 5 Å for side chain-backbone interactions.

The potential $\tilde{V}$ may be visualized as an energy function on a graph with one discrete site per amino acid. The graph has a connection between any two residues that are within the cutoff separation *R*_cutoff_ (Fig 3). The structure of this graph varies dynamically over the course of a simulation because the definition of neighboring residues depends on the backbone configuration {*b*_*i*_}. The potential varies smoothly as the backbone moves so long as the pairwise potential functions are continuous in the backbone coordinates. The potential $\tilde{V}$ is continuous despite the changing connections of the graph because the strength of the potential for each interaction approaches zero at *R*_cutoff_ just before the connection is eliminated from the graph. Problems such as this, with discrete potentials on an arbitrary graph, are extensively studied in both statistical mechanics (as variants of the Ising model) and machine learning (as undirected graphical models or Markov random fields) [7]. Below we adopt some well studied approximations from these fields to provide accurate and tractable methods for computing our coarse-grain potential $\bar{V}$.

Two approximations [7] are invoked to compute the free energy according to

$$\bar{V}=G^{\text{SC}}=-\text{log}\sum_{{\tilde{\chi }}_{1},\ldots ,{\tilde{\chi }}_{N}}e^{-v(\left\{{\tilde{\chi }}_{i}\right\})}.$$ (6)

The first approximation is to express the free energy *G*^SC^ in terms of the entropy and average energy of the Boltzmann ensemble where the entropy has been replaced by a mutual information approximation that ignores 3-residue and higher correlations,

$$\begin{array}{cc} G^{\text{SC}} & =\langle \bar{v}\rangle -S\\ & \approx \left\langle \bar{v}\right\rangle -S^{\text{approx}}, \end{array}$$ (7)

where $\langle \bar{v}\rangle$ and *S*^approx^ are defined in Eqs 8 and 9. We express the average energy and approximate entropy using the single-residue probabilities $p_{i}\left({\tilde{\chi }}_{i}\right)$ that residue *i* is in state ${\tilde{\chi }}_{i}$ in the Boltzmann ensemble of $\bar{v}$ and similarly for the joint probabilities $p_{ij}\left({\tilde{\chi }}_{i},{\tilde{\chi }}_{j}\right)$. Using *p*_*i*_ and *p*_*ij*_, the approximate energy and entropy are

$$\begin{array}{cc} \langle \bar{v}\rangle = & \sum_{i}\sum_{{\tilde{\chi }}_{i}}p_{i}\left({\tilde{\chi }}_{i}\right)v_{i}^{\left(1\right)}\left({\tilde{\chi }}_{i}\right)+\\ & \sum_{\begin{array}{c} i,j\\ \text{neighbors} \end{array}}\sum_{{\tilde{\chi }}_{i},{\tilde{\chi }}_{j}}p_{ij}\left({\tilde{\chi }}_{i},{\tilde{\chi }}_{j}\right)v_{ij}^{\left(2\right)}\left({\tilde{\chi }}_{i},{\tilde{\chi }}_{j}\right) \end{array}$$ (8)

$$\begin{array}{cc} S^{\text{approx}}= & \sum_{i}\sum_{{\tilde{\chi }}_{i}}p_{i}\left({\tilde{\chi }}_{i}\right)\left(-\text{log}\;p_{i}\left({\tilde{\chi }}_{i}\right)\right)-\\ & \sum_{\begin{array}{c} i,j\\ \text{neighbors} \end{array}}\sum_{{\tilde{\chi }}_{i},{\tilde{\chi }}_{j}}p_{ij}\left({\tilde{\chi }}_{i},{\tilde{\chi }}_{j}\right)\text{log}\frac{p_{ij}\left({\tilde{\chi }}_{i},{\tilde{\chi }}_{j}\right)}{p_{i}\left({\tilde{\chi }}_{i}\right),p_{j}\left({\tilde{\chi }}_{j}\right)}. \end{array}$$ (9)

We intend to minimize the approximate free energy in Eq 7 over all putative Boltzmann probability distributions for the side chain states $\left\{{\tilde{\chi }}_{i}\right\}$ (Step 3). Notice that only the single side chain probabilities *p*_*i*_ and joint side chain probabilities *p*_*ij*_ are required to compute the average energy and approximate entropy; we do not need the more complicated full joint probability distribution of the $\left\{{\tilde{\chi }}_{i}\right\}$ states for all side chains. In addition to the mutual information approximation of the entropy, we assume that any pair probability *p*_*ij*_ represents possible pair probabilities from a Boltzmann distribution, so that the only task is to minimize the free energy with respect to the pair probabilities. The only constraints imposed are that they must satisfy the obvious consistency conditions for probabilities,

$$p_{j}\left({\tilde{\chi }}_{j}\right)=\sum_{{\tilde{\chi }}_{i}}p_{ij}\left({\tilde{\chi }}_{i}\right)=\sum_{{\tilde{\chi }}_{k}}p_{jk}\left({\tilde{\chi }}_{k}\right)$$ (10)

$$\sum_{{\tilde{\chi }}_{i},{\tilde{\chi }}_{j}}p_{ij}\left({\tilde{\chi }}_{i},{\tilde{\chi }}_{j}\right)=1$$ (11)

$$p_{ij}\left({\tilde{\chi }}_{i},{\tilde{\chi }}_{j}\right)=p_{ji}\left({\tilde{\chi }}_{j},{\tilde{\chi }}_{i}\right).$$ (12)

However, the use of only conditions in Eqs 10–12 is insufficient to ensure that a joint probability distribution exists for all the variables consistent with the choices of *p*_*i*_ and *p*_*ij*_. As an explicit example,

$$p_{12}=p_{23}=(\begin{array}{ccc} 1/3 & 0 & 0\\ 0 & 1/3 & 0\\ 0 & 0 & 1/3 \end{array})$$ (13)

$$p_{13}=(\begin{array}{ccc} 1/9 & 1/9 & 1/9\\ 1/9 & 1/9 & 1/9\\ 1/9 & 1/9 & 1/9 \end{array})$$ (14)

obeys conditions Eqs 10–12 but is not representable by any probability distribution for the three residues. This aspect is a result of residue 1 being completely correlated to residue 2, and residue 2 being completely correlated to residue 3, but residues 1 and 3 being independent, which is mathematically impossible.

The issues of the approximation of the entropy and non-representability are potential concerns. However, we expect that they typically are not a large source of error given the comparable accuracy in predicting side chain rotamers as models employing full side chains. One limitation of these approximations is that the model cannot consider fully correlated side chain distributions. This limitation could be an issue for an allosteric switch which couples many side chain rearrangements with no accompanying backbone motion. As we represent the side chain with up to 6 possibly conformations, we are likely to capture a significant fraction of the conformation entropy for all but the longest side chains. Even then, each rotamer only contributes about 0.15 kcal/mol [8].

Accepting the two approximations for entropy and representability, the free energy becomes

$$G^{\text{SC}}\approx \underset{\left\{p_{i}\right\},\left\{p_{ij}\right\}}{\text{min}}\left(\left\langle \bar{v}\right\rangle -S^{\text{approx}}\right).$$ (15)

Thus, we now have a tractable approximation to free energy of the side chain. We can minimize that free energy using a self-consistent iteration technique called belief propagation (see Subsection **Belief propagation**). The iteration typically converges rapidly, often in 10-20 steps, to produce an approximation of the side chain free energy.

### Molecular dynamics simulations using the side chain free energy

In *Upside*, molecular dynamics simulations require calculations of the forces on the three backbone atoms (Step 4). The forces on all atoms are obtained from the derivatives of the potential computed according to $-\frac{d\bar{V}}{db_{i}}$. The forces on the O, H and bead are “pulled back” onto the three backbone atoms using the chain rule (Step 5). We take advantage of several terms being zero because the pair probabilities minimize the free energy,

$$\begin{array}{cc} \frac{dG^{\text{SC}}}{db_{k}} & =\frac{\partial G^{\text{SC}}}{\partial b_{k}}+\sum_{i}\frac{\partial G^{\text{SC}}}{\partial p_{i}}\frac{\partial p_{i}}{\partial b_{k}}+\sum_{\begin{array}{c} i,j\\ \text{neighbors} \end{array}}\frac{\partial G^{\text{SC}}}{\partial p_{ij}}\frac{\partial p_{ij}}{\partial b_{k}}\\ & =\frac{\partial G^{\text{SC}}}{\partial b_{k}}=\frac{\partial \langle \bar{v}\rangle }{\partial b_{k}}=\langle \frac{\partial \bar{v}}{\partial b_{k}}\rangle \\ & =\sum_{i}\sum_{{\tilde{\chi }}_{i}}p_{i}\left({\tilde{\chi }}_{i}\right)\frac{\partial v_{i}^{\left(1\right)}}{\partial b_{k}}\left({\tilde{\chi }}_{i}\right)+\\ & \;\sum_{\begin{array}{c} i,j\\ \text{neighbors} \end{array}}\sum_{{\tilde{\chi }}_{i},{\tilde{\chi }}_{j}}p_{ij}\left({\tilde{\chi }}_{i},{\tilde{\chi }}_{j}\right)\frac{\partial v_{ij}^{\left(2\right)}}{\partial b_{k}}\left({\tilde{\chi }}_{i},{\tilde{\chi }}_{j}\right) \end{array}$$ (16)

where $\frac{\partial G^{\text{SC}}}{\partial p_{i}}=\frac{\partial G^{\text{SC}}}{\partial p_{ij}}=0$ because *p*_*i*_ and *p*_*ij*_ are chosen to minimize *G*^SC^. The remaining simplifications occur because the partial derivative of *S*^approx^ with respect to the backbone coordinates *b*_*k*_ is zero (even though the total derivative $\frac{dS_{\text{approx}}}{db_{k}}$ is nonzero). While the underlying side chain interactions are pairwise additive and vanish outside the cutoff radius *R*_cutoff_, the free energy in Eq 7 is a many-body potential that can interact over arbitrary distances.

Since the approximate free energy due to the side chains is not a convex function of the probabilities, local minima may arise and impair the self-consistent iteration from finding the global minimum. To reduce the danger posed by the presence of local minima, calculations are begun from a carefully initialized state (see Subsection **Belief propagation** for details). Other self-consistent approximations exist for the side group free energy, such as tree-reweighted belief propagation [9], that are typically less accurate but always converge to the global minimum of their approximate free energy. Another limitation of the present approximation scheme arises when a bi-stable or multi-stable energy landscape is possible for the rotamer states. If well-separated and equally important minima are present for a single backbone configuration in the rotamer free energy surface, the probabilities only converge to a single minimum and thus underestimate the entropy of the side chains. While this does not appear to occur near the native well, we have not extensively searched for special backbone configurations that would result in bi-stable rotamer energies. The characterization of such problematic configurations, likely near free energy barriers, is left to future work.

### Bead locations and interactions

Paralleling the necessity of coarse-graining the rotamer states, side chain atoms themselves also require coarse-graining in order to obtain an inexpensive side chain model (Step 2). This reduction in the number of degrees of freedom is further justified since the atomic positions of the side chains are uncertain due to the discretization and aggregation of the rotamer states, meaning that there is little value in assigning precise positions for all atoms. We instead use a single oriented bead (3 spatial and 2 orientation coordinates) to represent each side chain (note that the direction is independent of the positions of the side chain atoms, e.g. in aromatic residues it could be the unit vector normal to the ring). The locations and directions of the side chain beads are updated during the optimization of the potential. The improvement in prediction accuracy from using optimized side chain positions rather than the static positions (e.g., side chain center-of-mass for different rotameric states) is surprisingly substantial.

**Fig 5:**
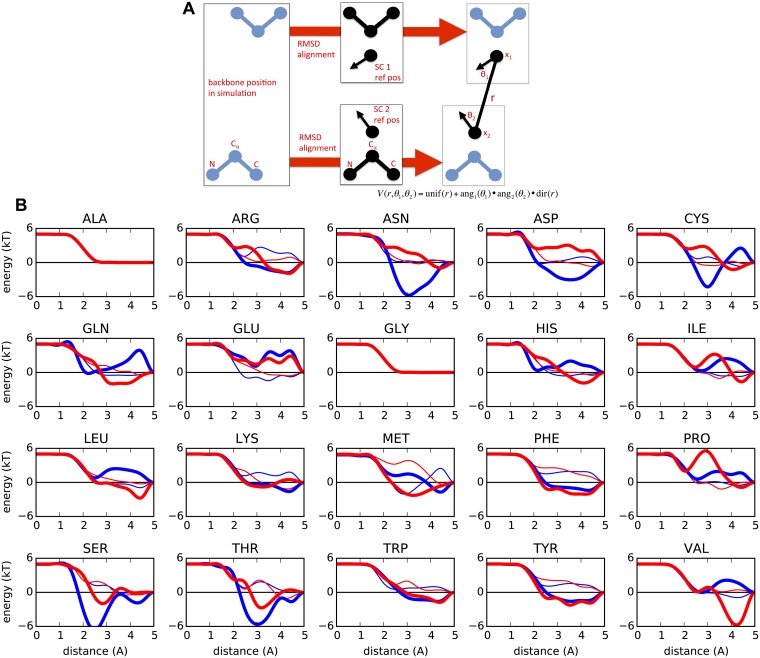
Coordinates and potentials used for side chain interactions. **Top**. For each residue, a reference backbone structure is aligned to the N, C_*α*_, and C atomic coordinates. This alignment creates a reference frame to establish the position and direction of the side chain bead. The two side chain beads *x*_1_ and *x*_2_ for a pair of residues establishes three coordinates, the distance *r* and angles *θ*_1_ and *θ*_2_. **Bottom**. Example of distance-dependent potential, unif(*r*_12_), after training, between the side chain and backbone residues. These interactions cutoff at 5 Å while the side chain-side chain interactions cutoff at 7 Å. The thin lines describe the *V*_radial_ of oxygen (red) and hydrogen (blue), and the thick lines describe *V*_radial_ + *V*_angular_ for the same interactions.

We use a combination of isotropic and directional interactions for each pair of interacting side chain or backbone (Fig 5). The isotropic interactions are primarily responsible for enforcing excluded volume, while the directional interactions typically reflect specific chemical interactions such as from polar groups or aromatic rings. Concretely, each interaction pair is described by positions *y*_1_ and *y*_2_ and directions *n*_1_ and *n*_2_. The separation *r*_12_ = |*y*_1_ − *y*_2_| and displacement unit vector *n*_12_ = (*y*_1_ − *y*_2_)/*r*_12_ are calculated. The form of the interaction is given by

$$\begin{array}{cc} V= & \kappa (V_{\text{radial}}\left(r_{12}\right)+\\ & \;{\text{ang}}_{1}\left(-n_{1}\cdot n_{12}\right)\text{ang}\left(n_{2}\cdot n_{12}\right)V_{\text{angular}}\left(r_{12}\right)), \end{array}$$ (17)

where *V*_radial_, ang_1_, ang_2_, and *V*_angular_ are smooth curves represented by cubic splines for increased flexibility (62 parameters total), rather than fixed functional forms such as a van der Waals 6-12 potential.

For side chain-side chain interactions, the *κ* prefactor is 1. But for side chain-backbone interactions, *κ* depends on the hydrogen bonding state of the backbone residue. This distinction reflects the observation that the presence of one hydrogen bond inhibits the formation of another due to competition for the single lone pair of electrons on the carbonyl oxygen that is available for hydrogen bonding. Specifically, the interaction between an amide proton or oxygen is given a hydrogen bond confidence score *f*, which is a number typically close to 0 for non-hydrogen bonded and 1 for hydrogen bonded residues. We set *κ* = 1 − *f* so that the interaction is only turned on for hydrogens or oxygens that are not already participating in a backbone-backbone hydrogen bond. The physical motivation is that the directional interaction primarily describes the effects of the dipole interactions, and the sum of the C = O and N–H dipoles have a vanishing dipole moment. While it is theoretically possible for the algorithm to carefully balance hydrogen and oxygen interactions that themselves cancel out on hydrogen bonded pairs, it is much easier to achieve a physically reasonable model if we enforce the zeroing of directional interactions with already hydrogen bonded pairs. The hydrogen bond distance and angular criteria are detailed in Subsection **Simulation details**.

The side chain-backbone interactions are needed to describe helix capping, where a side chain atom forms a hydrogen bond with an otherwise unsatisfied donor or acceptor at the end of helix. We have observed that a proper description of these capping effects is required to avoid helix fraying and inordinately long helices. Harper and Rose [10] have observed that N-terminal capping of a helix by side chains is more likely to be observed than is C-terminal capping by the side chain. This finding is consistent with our maximum-likelihood training (below), where side chain-amide hydrogen interactions are fit with stronger potentials (i.e., with higher confidence) than side chain-oxygen interactions. Harper and Rose also noted that hydrophobic residues play a strong role in helix capping by covering the exposed protein backbone at the ends of helices. To provide our model with the freedom to describe this effect, an additional side chain-backbone interaction is added with three beads, which could represent the possible hydrophobic character of the backbone. The location of the three beads are initialized from the reference position of N, C_*α*_, and C and are optimized with the rest of the parameters. For this interaction, *κ* = 1.

We have chosen to use 1- and 2-body potentials and have not employed 3- or 4-body potentials, in part due to difficulty of parameterizing the vast number of additional parameters. Similarly, higher order side chain entropy corrections would lead to a large increase in the computational cost of calculating the free energy, reducing the sampling ability and applicability to dynamics simulations. An alternative approach for introducing many-body effects and still maintaining the computational tractability, is to allow the pair interactions to depend on discrete parameters, such as the rotomer index used in the present study.

### Maximum-likelihood training

The side chain model is trained by the maximum-likelihood principle. Specifically, we determine the set of parameters that maximizes the log probability of the true side chain states ${\tilde{\chi }}_{p}$ in the Boltzmann ensemble of all possible side chain states $\tilde{\chi }$ for the fixed backbone positions *X*_*p*_ for each protein *p*.

$$p({\tilde{\chi }}_{p})=\frac{e^{-V({\tilde{\chi }}_{p})}}{\sum_{\tilde{\chi }}e^{-V(\tilde{\chi })}}$$ (18)

$$-\text{log}\;p({\tilde{\chi }}_{p})=V\left({\tilde{\chi }}_{p}\right)+\text{log}(\sum_{\tilde{\chi }}e^{-V(\tilde{\chi })})$$ (19)

$$=V\left({\tilde{\chi }}_{p}\right)-G^{\text{SC}}$$ (20)

$$=E_{\text{gap}}.$$ (21)

The evaluation of *E*_gap_ requires the evaluation of the free energy of the side chains, a quantity that is intractable to calculate exactly. Fortunately, our side chain energy Eq 15 approximates the true side chain free energy *G*^SC^ that appears in Eq 20. Furthermore, the expression for the parametric derivative Eq 16 allows for gradient descent optimization to minimize the average gap energy.

### Training set

The side chain packing interaction is trained using a large, non-redundant collection of crystal structures from the PDB with 50–500 residues and resolution less than 2.2 Å. From a training set of protein structures, we extract the sequences *s*_*p*_, backbone trace positions *X*_*p*_, and true coarse-grained side chain states ${\tilde{\chi }}_{p}$ for each protein *p*. The proteins are further filtered using PISCES [11] so that all pairs of proteins have sequence similarity less than 30%. Non-globular structures in the dataset are removed, as we suspect that the side chain packing of these structures is more strongly influenced by other chains in the crystal structures. We define non-globular structures as outliers in the linear relationship between log(*N*_res_) and log(*R*_*g*_); the outliers are identified using the RANSAC algorithm [12]. After filtering, 6255 chains remained, containing approximately 1.4 million residues.

### Belief propagation

This subsection contains a brief description of the equations used to implement belief propagation for the side chain free energies. Given 1-residue energies $v_{i}\left({\tilde{\chi }}_{i}\right)$ and 2-residue energies $v_{ij}\left({\tilde{\chi }}_{i},{\tilde{\chi }}_{j}\right)$, we seek probabilities $p_{i}\left({\tilde{\chi }}_{i}\right)$ and $p_{ij}\left({\tilde{\chi }}_{i},{\tilde{\chi }}_{j}\right)$ to minimize the free energy in Eq 15.

It is helpful to first understand the intuition behind the belief propagation process. We seek a consistent set of 1- and 2-side chain probabilities for the residues compatible with the interaction potential Eq 5. The probability of each residue state ${\tilde{\chi }}_{i}$ for residue *i* is determined by two factors. The first factor is the 1-residue energy $v_{i}\left({\tilde{\chi }}_{i}\right)$ that would determine the probabilities exactly in the absence of interactions. The second factor is consistency with the side chain states of the residues in contact with residue *i*, where consistency is determined by the potentials $v_{ij}\left({\tilde{\chi }}_{i},{\tilde{\chi }}_{j}\right)$. Belief propagation optimizes these factors to minimize the approximate free energy Eq 15 as derived in reference [13]. The iteration is described more formally below, including a damping term λ to suppress oscillations during the self-consistent iteration.

For 1-residue beliefs, we define $b_{i}^{r}\left({\tilde{\chi }}_{i}\right)$ to be the round *r* “belief” that the *i*-th residue is in state ${\tilde{\chi }}_{i}$. For the 2-residue beliefs, we have two beliefs for each pair of interacting residues (i.e. any pair of residues that interact in any rotamer states). Define $b_{ij}^{r}\left({\tilde{\chi }}_{j}\right)$ to be the round *r* belief for the residue pair (*i*,*j*) that residue *j* is in state ${\tilde{\chi }}_{j}$. The belief $b_{ji}^{r}\left({\tilde{\chi }}_{i}\right)$ is defined similarly.

To initialize the algorithm at round 0, we take

$$b_{i}^{0}\left({\tilde{\chi }}_{i}\right)=e^{-v_{i}\left({\tilde{\chi }}_{i}\right)}$$ (22)

$$b_{ji}^{0}\left({\tilde{\chi }}_{i}\right)=\sum_{{\tilde{\chi }}_{j}}e^{-v_{ij}\left({\tilde{\chi }}_{i},{\tilde{\chi }}_{j}\right)}b_{j}^{0}\left({\tilde{\chi }}_{j}\right).$$ (23)

We compute the round *r* + 1 beliefs from the round *r* beliefs according to the following equations.

$$b_{ji}^{r+1}\left({\tilde{\chi }}_{i}\right)=\sum_{{\tilde{\chi }}_{j}}e^{-v_{ij}\left({\tilde{\chi }}_{i},{\tilde{\chi }}_{j}\right)}\frac{b_{j}^{r}\left({\tilde{\chi }}_{j}\right)}{b_{ij}^{r}\left({\tilde{\chi }}_{j}\right)}$$ (24)

$$b_{i}^{r+1}\left({\tilde{\chi }}_{i}\right)=\lambda b_{i}^{r}\left({\tilde{\chi }}_{i}\right)+\left(1-\lambda \right)\frac{e^{-v_{i}\left({\tilde{\chi }}_{i}\right)}\prod_{j}b_{ji}^{r+1}\left({\tilde{\chi }}_{i}\right)}{\sum_{{\tilde{\chi }}_{i}}e^{-v_{i}\left({\tilde{\chi }}_{i}\right)}\prod_{j}b_{ji}^{r+1}\left({\tilde{\chi }}_{i}\right)}$$ (25)

The products in Eq 25 should be understood as taken only over residues *j* that interact with residue *i*. The damping constant λ suppresses oscillatory behavior that hinders convergence (λ = 0.4 is used in the present work). The equations are iterated until $|b_{i}^{r+1}\left({\tilde{\chi }}_{i}\right)-b_{i}^{r}\left({\tilde{\chi }}_{i}\right)|<0.001$ for all residues *i* and states ${\tilde{\chi }}_{i}$.

From the converged beliefs $b_{i}\left({\tilde{\chi }}_{i}\right)$ and $b_{ij}\left({\tilde{\chi }}_{j}\right)$, we can compute the marginal probabilities

$$p_{i}\left({\tilde{\chi }}_{i}\right)=b_{i}\left({\tilde{\chi }}_{i}\right)$$ (26)

$$p_{ij}\left({\tilde{\chi }}_{i},{\tilde{\chi }}_{j}\right)=\frac{\frac{b_{i}\left({\tilde{\chi }}_{i}\right)}{b_{ji}\left({\tilde{\chi }}_{i}\right)}e^{-v_{ij}\left({\tilde{\chi }}_{i},{\tilde{\chi }}_{j}\right)}\frac{b_{j}\left({\tilde{\chi }}_{j}\right)}{b_{ij}\left({\tilde{\chi }}_{j}\right)}}{\sum_{{\tilde{\chi }}_{i},{\tilde{\chi }}_{j}}\frac{b_{i}\left({\tilde{\chi }}_{i}\right)}{b_{ji}\left({\tilde{\chi }}_{i}\right)}e^{-v_{ij}\left({\tilde{\chi }}_{i},{\tilde{\chi }}_{j}\right)}\frac{b_{j}\left({\tilde{\chi }}_{j}\right)}{b_{ij}\left({\tilde{\chi }}_{j}\right)}}.$$ (27)

The free energy of the model is obtained by using the marginal probabilities above in Eq 15.

### Simulation details

The simulations are run with *Upside*. The replica exchange temperatures are 0.500, 0.532, 0.566, 0.600, 0.636, 0.672, 0.709, 0.748, 0.787, 0.828, 0.869, 0.912, 0.955, and 1.000. The Ramachandran potential uses the NDRD TCB coil library [14]. The backbone hydrogen bond interaction uses both distance and angle criteria to determine hydrogen bonds. The H-O bond distance interaction starts at approximately 1.4 Å and ends at 2.5 Å. Both the N-H-O and H-O-C criteria half-heights are at approximately 47 degrees off of co-linear.

We use Verlet integration with a time step of 0.009 units. We use the random number generator Random123 [15] to implement the Langevin dynamics with a thermalization time scale of 0.135 time units. The thermalization time scale (related to Langevin friction) is chosen to maximize the effective diffusion rate of chains while effectively controlling simulation temperature. As Langevin dynamics with any friction coefficient produces the same Boltzmann ensemble, we chose to maximize equilibration of our system rather than attempt to match a solvent viscosity.

The cutoff radius for side chain-side chain interactions is 7Å, and the cutoff radius for side chain-backbone interactions is 5Å. The distance splines are zero-derivative-clamped cubic splines with a knot spacing of 0.5Å. The angular splines have a knot spacing of 0.167 in cos*θ*, which ranges over [−1, 1].

### Optimization details

The Adam optimizer [16], a popular algorithm to optimize noisy objective functions, is used to minimize the energy gap. This optimizer is convenient because it automatically adjusts the gradient descent step size for each parameter according to the typical scale of the gradient in that dimension. This rescaling is important because spline coefficients at large radii tend to have much larger gradient magnitudes than parameters at small radii.

We use the following settings for the Adam optimizer: minibatch size of 256 proteins, *α* = 0.03, *β*_1_ = 0.90, *β*_2_ = 0.96, *ϵ* = 10^−6^. Positivity constraints on the angular coefficients are enforced by a exponential transform. The regularization integrals over all space are approximated by sums at the knot locations of the radial and angular splines.

A regularization penalty is added to the maximum-likelihood optimization that encourages smoothness of the potential. This penalty also reduces the validation error of the training. The regularization penalties chosen are

$$\sum_{i}{\left(2c_{i}^{\text{unif}}-c_{i-1}^{\text{unif}}-c_{i+1}^{\text{unif}}\right)}^{2}$$ (28)

$$\sum_{i}{\left(c_{i}^{\text{dir}}\right)}^{2}$$ (29)

$$\sum_{i}{\left(c_{0}^{\text{unif}}-\left(5\;k_{\text{B}}T\right)\right)}^{2}$$ (30)

The penalty Eq 28 encourages a small second derivative for the isotropic (unif) term, while the penalty Eq 29 minimizes the size of the directional interactions. Finally, the penalty Eq 30 ensures a strong steric core for interactions.

The derivative calculations needed for regularization and coordinate transforms are handled with the Tensorflow framework [17].

### Optimized mapping to coarse states

The *χ*-angles for the side chains are partitioned into discrete states in an optimized manner (Fig 4). The NDRD rotamer library [18] provides a set of approximate discrete states for each residue type according to their frequencies of occurrence in a non-redundant set of high resolution protein structures in the PDB. However, the number of rotamer states in the NDRD library can be quite large. For instance, naively using all 81 rotamers for each arginine means that computing the pair interaction *v*_*i*, *j*_ for two arginines would require computing 81^2^ = 6561 energy values. Consequently, instead of using all possible rotamer states, several NDRD rotamer states are combined into 3–6 coarse-grained rotamer states for the sake of manageable computational cost.

We choose to aggregate the rotamer states of the side chain to minimize the positional uncertainty of side chain atoms in each state. A search over all possible aggregations is conducted to find the aggregation that provides the smallest possible error. More formally, the NDRD rotamer library [18] is used to define the atomic positions $x_{ij}^{f}\left(\phi ,\psi \right)$, where *i* is the atom (such as C_*β*_), *j* is the coordinate (*x*, *y*, or *z*), and *f* is the fine-grained rotamer state. Each rotamer state has a probability *p*^*f*^(*ϕ*, *ψ*) specified in the NDRD library from frequencies in the PDB for each fine-grained rotamer state as a function of the backbone dihedral angles (*ϕ*, *ψ*). Each fine-grained state *f* may belong to exactly one coarse-grained state *c* (i.e. the *c* states form a partition of the *f* states). Given the choice of a coarse-grained state *c*, an average is performed over the fine-grained atomic positions, and sum is taken over the probabilities of all fine-grained states *f* grouped into *c* according to the prescription,

$$q^{c}\left(\phi ,\psi \right)=\sum_{f\in c}p^{f}\left(\phi ,\psi \right)$$ (31)

$$y_{ij}^{c}\left(\phi ,\psi \right)=\frac{1}{q^{c}\left(\phi ,\psi \right)}\sum_{f\in c}p_{ij}^{f}\left(\phi ,\psi \right)\;x_{ij}^{f}\left(\phi ,\psi \right),$$ (32)

where *q*^*c*^ is the coarse-grained probability and $y_{ij}^{c}$ is the coarse-grained atomic position.

The error incurred by coarse-graining is defined as the variance of the atom positions within each coarse-grained state, weighted by the frequency of occurrence of the coarse-grained state in the PDB. Specifically, the error *σ*^2^(*ϕ*, *ψ*) is defined as,

$$\sigma^{2}\left(\phi ,\psi \right)=\sum_{f}\frac{p^{f}\left(\phi ,\psi \right)}{N_{\text{atom}}}\sum_{ij}{\left(x_{ij}^{f}\left(\phi ,\psi \right)-y_{ij}^{c(f)}\left(\phi ,\psi \right)\right)}^{2},$$ (33)

where *N*_atom_ is the number of atoms in the side chain and *c*(*f*) is the coarse-grained state *c* that contains the fine-grained state *f*. The error depends implicitly on the state decomposition *c*(*f*) and measures the deviation of the atoms within each state. This error favors the fine-grained states *f* that occur with higher frequency in the PDB.

The division of fine-grained states into coarse-grained states is restricted for simplicity to be independent of the Ramachandran angles for the residue,

$$\sigma^{2}=\int p^{\text{Rama}}\left(\phi ,\psi \right)\;\sigma^{2}\left(\phi ,\psi \right)\;d\phi \;d\psi ,$$ (34)

where *p*^Rama^(*ϕ*, *ψ*) is the frequency of each Ramachandran angle taken from the PDB coil library. Note that this error term depends implicitly on the decomposition *c*(*f*) and weights for the (*ϕ*, *ψ*) pairs according to their frequencies in the coil library.

An optimal coarse-grained representation of the side chain rotamer states is obtained by minimizing *σ*^2^ for each residue type over all partitions *c*(*f*). We force the coarse-graining *c*(*f*) to obey a few conditions, essentially to make sure that *c*(*f*) is easily interpretable in terms of *χ*_1_ and *χ*_2_ as well as limiting the number of possibilities that must be checked by the brute-force minimization. In particular, the mapping from coarse states back to *χ*_1_ rotamer states is unambiguous because no single coarse state contains two different *χ*_1_ rotamer states. We impose the following conditions:

1. *c*(*f*) depends only on the *χ*_1_ and *χ*_2_ rotamer states of *f* (i.e. if *f*_1_ and *f*_2_ states differ only in their *χ*_3_ or *χ*_4_ states, then *c*(*f*_1_) = *c*(*f*_2_)).
2. Each coarse state *c* must contain only a single *χ*_1_ state but may contain multiple distinct *χ*_2_ states for that *χ*_1_ state.
3. Each coarse state *c* must contain a contiguous range of *χ*_2_ values. This greatly reduces the number of possible coarse-grainings for residues with non-rotameric *χ*_2_ angles like asparagine.

We optimize the decomposition of the coarse-grained state *c*(*f*) by completely enumerating all possible decompositions into coarse-grained states that satisfy the three conditions above and contain no more than six coarse states.

### Backbone parameters

The backbone atoms interact with a soft-sphere repulsion at approximately 3 Å interatomic distance. The equilibrium distances of the N–C_*α*_, C_*α*_–C, and C–N bonds are 1.453 Å, 1.526 Å, and 1.300 Å, respectively. The backbone angles are restrained at their ideal values (109.5° and 120°).

## Results

### Packing accuracy

The accuracy of the results are computed in two ways. The first measure computes the accuracy of the one-residue probabilities at predicting the *χ*_1_ states of the protein. This quantity is the traditional accuracy measure for side chain packing algorithms. The second measure is the quality of the ensemble, obtained by computing the difference (*E*_gap_) between the free energy of the side chain system and the potential energy of the crystallographic rotamer configuration (Eq 20). For a highly accurate side chain ensemble, we would expect that the crystal configuration would be a high probability state in the ensemble and thus the *E*_gap_ would be small. This energy gap is minimized by the maximum-likelihood training. The two accuracy measures are typically linearly related for the side chain models we consider.

**Fig 6:**
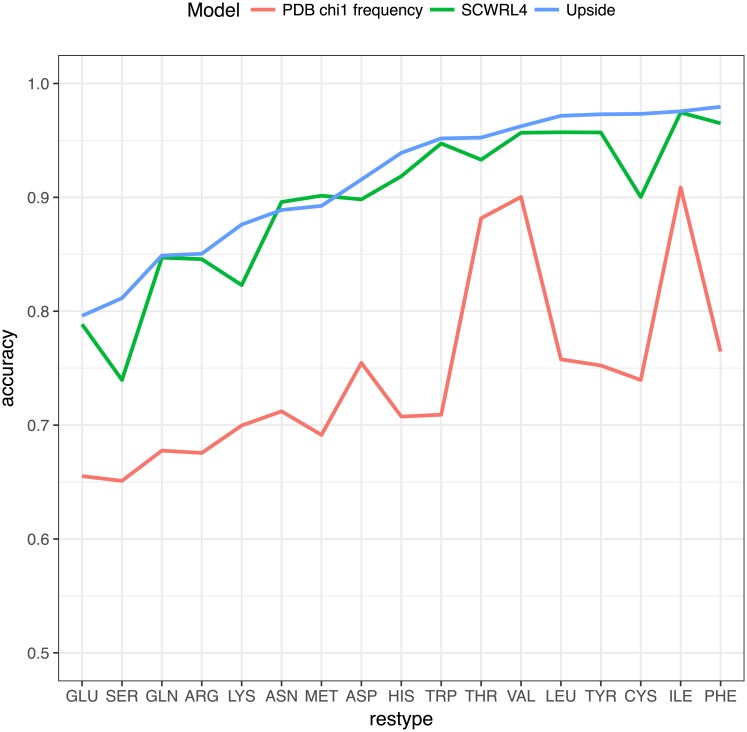
Comparison of *χ*_1_ prediction accuracy for *Upside* and SCWRL4, ordered by *Upside* accuracy. The “PDB *χ*_1_ frequency” line represents the accuracy of the NDRD rotamer library without any interactions; this library is used in both *Upside* and SCWRL4.

**Fig 7:**
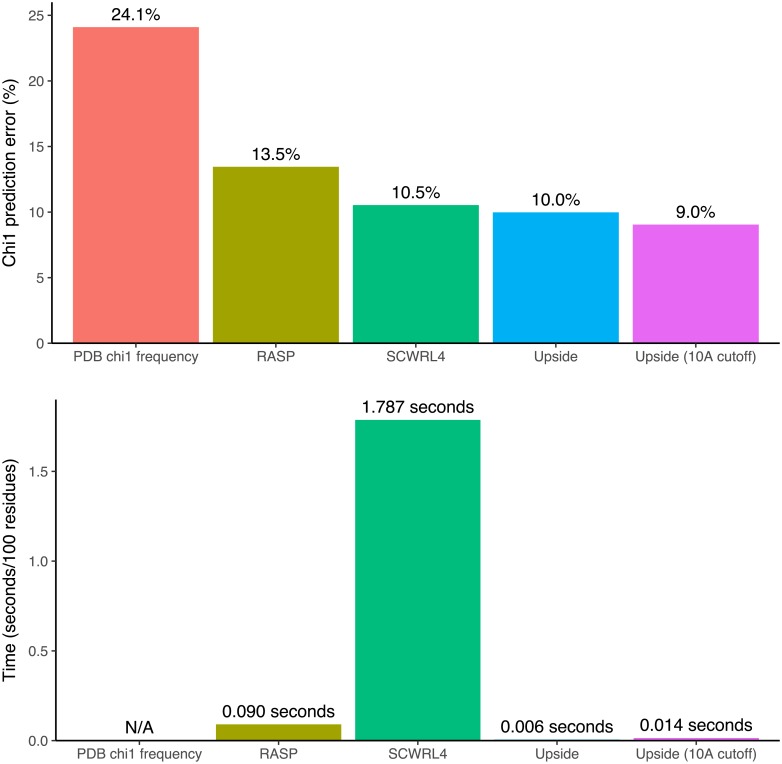
Comparison of the accuracy of predicting side chains as well as cpu running time. For all programs, time spent reading the protein structure and writing the results is excluded from the running time to focus on the cost of solving for the side chain positions. For Upside (10 Å cutoff), all side chain interactions with backbone or other side chains are cutoff at 10 Å.

To compare to modern side chain prediction methods, we benchmark against SCWRL4 [3] on its training and validation set of side chains conformations (Fig 6), as well as the RASP algorithm [6] for rapid side chain packing (Fig 7). Since the *Upside* model lacks full side chains, we use the most likely *χ*_1_ rotamer state according to the 1-residue marginal distributions $p_{i}\left({\tilde{\chi }}_{i}\right)$. As per SCWRL4’s validation procedure, the lowest confidence side chains are excluded (bottom 25^th^ percentile in electron density). To avoid biasing the comparison toward *Upside*, the SCWRL4 training set is split so that 20% of the proteins are withheld for measuring accuracy, while the rest are used for maximum-likelihood training. The accuracy metric chosen is to calculate the fraction of side chains for which the *Upside* or SCWRL4 predicted *χ*_1_ rotamer state agrees with the crystallographic conformation. The residues alanine, glycine, and proline are excluded from the comparison.

Comparison of *χ*_1_ prediction accuracy for *Upside* and SCWRL4, ordered by *Upside* accuracy. The “PDB *χ*_1_ frequency” line represents the accuracy of the NDRD rotamer library without any interactions; this library is used in both *Upside* and SCWRL4. *Upside* is accurate, predicting the correct *χ*_1_ rotamer 91.0% of the time using 10 Å cutoffs, which is better than SCWRL4 [3] or RASP [6]’s values of 89.5 and 86.5%, respectively (Figs 6 and 7). Additionally, *Upside* predicts side chains 16 times faster than the speed-optimized RASP and 300 times faster than accuracy-optimized SCWRL4. This very fast calculation enables *Upside*’s side chain model to be viable in the inner loop of molecular dynamics simulations, as discussed in the next section.

We examined the importance of various interactions in *Upside* by recalculating the change in accuracy upon their removal. For example in Table 1, we calculated the decrease in performance after retraining our parameters on the PDB-based test set after removing one or more energy terms. One can see that using only repulsive interactions causes a 3.8% drop in side chain prediction accuracy compared to the full model, which quantifies the importance of the attractive interactions.

**Table 1:** Accuracy of predicting *χ*_1_ for the SCWRL4 data set.

Energy terms used	Accuracy change (%)	Δ*E*_gap_ (*k*_B_*T*)
10Å cutoffs	+0.7	-0.028
Full model	0.0	0.000
No H/O interactions	-0.6	0.013
No N,C_*α*_,C beads	-2.3	0.040
*ϕ*, *ψ*-independent *V*(*χ*)	-3.3	0.004
Isotropic only	-3.5	0.080
Repulsive only	-3.8	0.060
Side chain—side chain only	-3.8	0.067
Side chain—backbone only	-6.1	0.125
No interactions	-13.7	0.435

The significance of various components of the model reflect the decrease in accuracy for their removal and retraining on the entire training set. The parameters are separately optimized for each row of the table so that each *E*_gap_ represents the best achievable for the indicated functional form. Training was redone for each energy function and the ensuing accuracy was reevaluated. Results shown are for 20% of the SCWRL4 data set withheld for testing purposes. Note that these predictions are based on single-chain structures, so they differ slightly in accuracy from the predictions on all-chain structures reported in Fig 6.

### Molecular dynamics simulations

To test the suitability of adapting the side chain packing model to study protein dynamics, Langiven dynamics folding simulations were run on small, fast-folding proteins (Table 2) using a standard Verlet algorithm that obeys detailed balance and conserves energy. The parameters obtained from the maximum-likelihood training are optimized for side chain packing for a set of fixed, native backbones, which is not the situation during the simulations where the backbone moves. In the limit that the model is flexible enough to describe the true side chain interactions and there are unlimited training data, the maximum-likelihood method should recover the true side chain interactions. Even without having the true form of the side chain interaction, the maximum-likelihood parameters assign high probability to the observed rotamer states, thereby providing evidence that it includes a significant portion of the underlying physics, and thus may be viable for use in molecular dynamics simulations.

**Table 2:** Sequences of proteins for molecular dynamics simulations.

Name	PDB ID	Length	Sequence
alpha3d	2a3d	73	MGSWAEFKQRLAAIKTRLQALGGSEAELAA FEKEIAAFESELQAYKGKGNPEVEALRKEA AAIRDELQAYRHN
BBL	2wxc	47	GSQNNDALSPAIRRLLAEWNLDASAIKGTG VGGRLTREDVEKHLAKA
homeodomain	2p6j	52	MKQWSENVEEKLKEFVKRHQRITQEELHQY AQRLGLNEEAIRQFFEEFEQRK

Since the maximum likely-hood training was conducted on proteins with fixed backbones, to create a reasonable model for dynamics, a basic Ramachandran potential, backbone springs and sterics, and a hydrogen bond energy, are added to the side chain model (see Subsection **Simulation details**). The Ramachandran nearest-neighbor dependent potential is derived from a coil library [14] as a statistical potential. The hydrogen bond enthalpy is varied to find the maximum accuracy. Note that because alanine and glycine have no side chain rotamer states, and hence no training to match the native *χ*-angles is feasible, the ALA-ALA, ALA-GLY, and GLY-GLY potentials are completely determined by the regularization. Interactions of ALA and GLY with other residue types are optimized, however, as rotamer states of the other residues provide information on the ALA-X and GLY-X interactions.

The hydrogen bond term does not play an explicit role in the packing optimization as the backbone and associated hydrogen bonds remain fixed during side chain placement. Hence, this term is not trained during the maximum likelihood procedure for the side chain positions. To assign an energy to the hydrogen bond term, it was manually varied for the best simulation accuracy. This term is the only parameter manually optimized for simulation accuracy.

**Fig 8:**
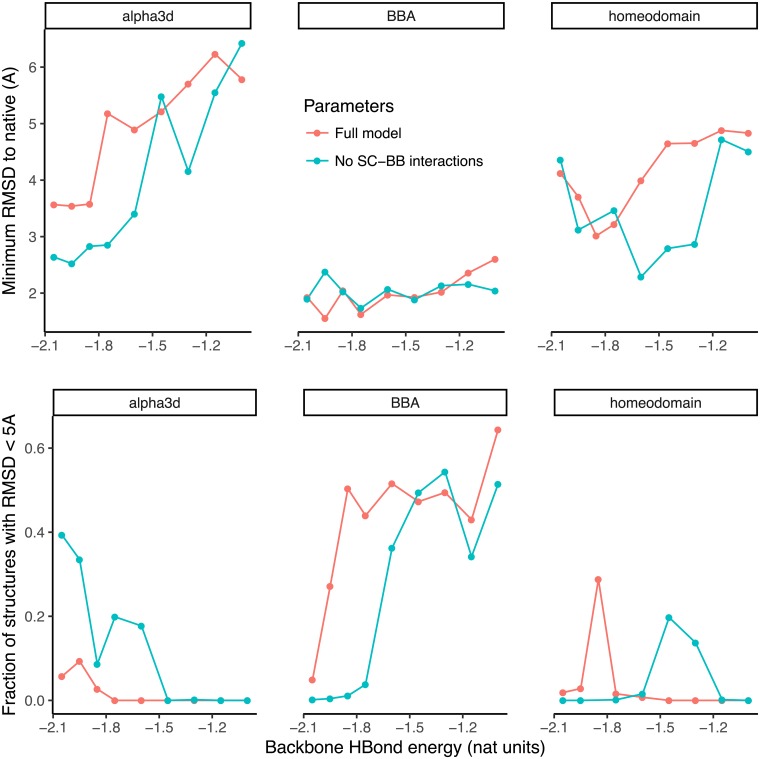
Accuracy of MD simulations for three proteins at variable backbone hydrogen bond strength. Results with and without side chain-backbone interactions are presented.

**Fig 9:**
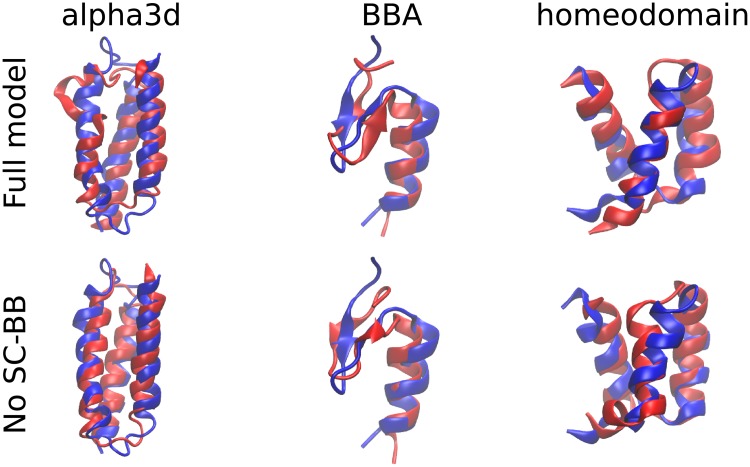
Closest structure to native protein (lowest C_*α*_-RMSD) at optimal hydrogen bonding strength, with and without backbone-side chain hydrogen bonds. For alpha3D, BBA and homeodomain with backbone-side chain hydrogen bonds, the optimal hydrogen bond strength and lowest C_*α*_-RMSD are -2.0, -2.0 and -1.9 RT, and 3.6, 1.7 and 3 Å, respectively. Without these hydrogen bonds, the corresponding values are -2.0, -1.8 and -1.6 RT; and 2.7, 1.8, and 2.5 Å. Blue is the native structure and red is the simulation.

Simulations were run on four small proteins. We obtained commendable results on three, alpha3D, BBA and a homeo domain, but not on a WW domain. We manually scanned through different hydrogen bond strengths to find an optimal for folding accuracy (Fig 8). For the three successful proteins, sub-3 Å structures were obtained in under two cpu-days (lowest C_*α*_-RMSD, Fig 9). Although performance depended on hydrogen bond strength, a single value of -1.8 units produced near-optimal results across the three proteins. The removal of side chain-backbone hydrogen bonds had a surprisingly small and sometimes even a positive effect on accuracy. Evidently for these proteins, helix capping signals are not important. More proteins and better training procedures are needed to investigate the generality of this finding. Overall, these results demonstrate that our model has the capability of folding proteins on the cpu-day time-scale. In the companion paper, we investigate the models potential for folding proteins when the energy function is trained for this purpose.

## Discussion

We have demonstrated a fast, principled method to coarse-grain discrete side chain states and create a smooth backbone potential. This procedure results in a considerable decrease in computational time as it removes the side chain rattling and friction normally associated with a polypeptide chain moving in a condensed state. This tracking and instantaneous equilibration of the side chains is analogous to the instantaneously-equilibrated electronic degrees of freedom with respect to the nuclear motions employed in the adiabatic Born-Oppenheimer approximation [19]. Motions are calculated only for three heavy backbone atoms, yet the model contains considerable structural detail including hydrogen bonds involving both the backbone and side chains. Further, we have presented both a maximum likelihood procedure to obtain a physically-reasonable potential from the side chain packing of X-ray structures and a tunable discretization of the rotamer states. The resulting method is capable of rapid molecular dynamics of protein structures with comendable accuracy considering the computational speed.

*Upside* is a coarse-grain model, and hence, certain details will be approximate especially for the unfolded state. However, our side chain energies include a rotameric term reflecting the intrinsic *χ*_1_ preference so we anticipate that our predicted *χ*_1_ distribution will be reasonable, especially for side chains typically found on the surface.

### Comparison to previous work

We highlight several works related to the major features of our model, including molecular dynamics on three atoms but with a dynamic ensemble of side chains, optimized discretization of the side chain states to best represent the protein interactions in the coarse-grained model, a potential with optimized and state-dependent bead locations and orientations, training a protein interaction model for folding using side chain packing accuracy, and a side chain model with an explicit side chain entropy.

A large body of work, exemplified by SCWRL4 [3], has studied the prediction of side chain configurations by discrete rotamer states (Figs 6 and 7). SCWRL4 achieves approximately 90% *χ*_1_ accuracy for predicting the most likely rotamer states by minimizing the energy that combines observed rotamer state frequencies and an atomic interaction model [3]. A variety of algorithms have been developed to solve for the highest probability side chain states given the pair interaction values [20, 21]. Kamisetty et al.[22] have worked on scoring protein interaction complexes using a self-consistent approximation to the side chain interactions. Earlier simulation work by Koehl and Delarue [23] use 1-residue mean field techniques to approximate ensembles of side chain conformations but fail to account for the pairwise correlations of the side chain rotamer states. All of these works use atomically-detailed descriptions of the side chains paired with simple or molecular dynamics interaction terms. Their highly detailed side chains with many *χ*-angles for each residue make it difficult to perform calculations sufficiently fast for folding simulations, and the use of existing interactions (instead of a newly-trained interaction model) makes it difficult to reduce detail to increase the computational speed. There has also been extensive work in reconstructing backbone positions from side chain beads [24] in lattice models, but these models do not perform a proper summation over possible rotamer states.

RASP [6] is side chain modeling program designed to significantly improve the speed of side chain packing while achieving comparable accuracy. The authors use careful selection of the most important energy terms as well as employing clash-detection to guide the optimization of the side chain conformations. A recent method, OSCAR-o [4], employs a genetic algorithm for swapping low energy side chain conformations. Oscar utilizes a distance- and orientational dependent energy function that is optimized for side chain packing accuracy [5], similar to *Upside*’s side chain potential.

Kihara and coworkers [25] conducted a thorough study of side chain accuracy in different environments. They found that OSCAR-o and the speed optimized OSCAR-star performed better than the other methods including SCWRL4, RASP and Rosetta, having a mean prediction accuracy of 88% versus 85, 85 and 83% accuracy, respectively, on their monomeric test set. Since *Upside*’s accuracy is very similar to SCWRL4, we infer that *Upside*’s performance does not quite match the OSCAR methods. However, the timing comparison presented indicates that *Upside* should be 4 and 2.5 orders of magnitude faster than OSCAR-o and OSCAR-star, respectively.

There have also been a large number of coarse-grained techniques that use a variety of non-isotropic potentials for reduced side chain interactions. One of the most successful is the coarse-grained united residue model (UNRES) [26]. The model also uses statistical frequencies to determine the positions of the side chains but it emphasizes the parameterization of the coarse-grained model from physics-based calculations instead of statistical information. Though the potential form (Gay-Berne) used in UNRES is quite different from our work, UNRES also uses non-isotropic side chain potentials [27].

Similar to our work, Dama, Sinitskiy, et al. [28] investigate mixed continuous-discrete dynamics, where the states of molecules jump according to a discrete Hamiltonian. Their method differs from our work in a number of important ways: the authors use discrete jumps in state instead of a free energy summation over all states that we employ; they do not optimize the rotamer states as we do; and they train parameters from force matching of molecular dynamics trajectories rather than from the statistical analysis of experimental data as we employ.

### Combination of *Upside* with other methodologies

The reason that *Upside* is both faster and has similar accuracy than competing methods at side chain packing is that *Upside* shifts the complexity of the *χ*_1_-prediction problem. Traditional side chain prediction uses a detailed configuration space of all rotamers and side chain atoms but simple interaction forms with few parameters. *Upside* uses a coarse configuration space with only a single directional bead per residue but a complex and well-optimized set of parameters consisting of over 10,000 jointly-optimized parameters (trained on approximately 500,000 residues). *Upside* demonstrates that *χ*_1_ rotamers can be predicted with state-of-the-art accuracy without needing to examine fine-grained atomic packing. Additionally, the side chains in *Upside* are represented as a Boltzmann ensemble whose 1-residue marginal probabilities are used to predict *χ*_1_ instead of predicting *χ*_1_ using the lowest energy configuration. This approach allows for the natural consideration of side chain entropy and conformational variability. Creating a Boltzmann ensemble over rotamer states also allows exact, continuous forces to be defined for the approximate ensemble, enabling molecular dynamics using potential energies already validated to represent the physics of side chain packing.

A natural question is whether the strengths of SCWRL4 and this algorithm may be combined. There are two reasons to believe that such a combination would be fruitful. The first reason is that when *Upside* and SCWRL4 predict the same *χ*_1_ rotamer, the prediction is 95.4% accurate, substantially more accurate than either program alone. This suggests *Upside* and SCWRL4 provide independent information about the side chain conformations and hence, combining both approaches should produce a substantially better packing model. The second reason that *Upside* and SCWRL4 may be combined is that *Upside* provides probability functions as its outputs, rather than just the minimum energy conformation as in SCWRL4. The underlying SCWRL4 single-rotamer energies could be augmented with $-\lambda \text{log}\;p_{Upside}\left(\tilde{\chi }\right)$. For an appropriately determined λ, this should incorporate some of *Upside*’s information directly into SCWRL4, increasing SCWRL4’s accuracy. Alternatively, SCWRL4’s detailed but simple energy function could be augmented by an *Upside*-style coarse-grained function, possibly with additional maximum-likelihood tuning.

### Conclusion

For side chain packing applications, *Upside* accurately and rapidly predicts of *χ*_1_ rotamer states and their probabilities. *Upside* takes advantage of these two features for dynamics applications, and it shows considerable promise as a route to accurate and inexpensive molecular simulation. New training techniques are being developed to directly optimize the backbone accuracy of the *Upside* model. In the companion paper, we present results using new training methods that indicate that we are able to achieve dramatic improvements in the accuracy of *de novo* folding while preserving the rapid folding properties for a variety of proteins. We expect that our belief-propagated side chains will serve as an excellent basis for new methods in protein simulations.

Source code for side chain packing and molecular simulations can be obtained from https://github.com/sosnicklab/upside-md, and the results of this paper can be reproduced using the version tagged sidechain_paper.
